# Implementation and Optimization of the Embedded Cluster Reference Interaction Site Model with Atomic Charges

**DOI:** 10.1021/acs.jpca.1c07904

**Published:** 2022-04-08

**Authors:** Ádám Ganyecz, Mihály Kállay

**Affiliations:** Department of Physical Chemistry and Materials Science, Budapest University of Technology and Economics, Budapest P.O. Box 91, H-1521 Hungary

## Abstract

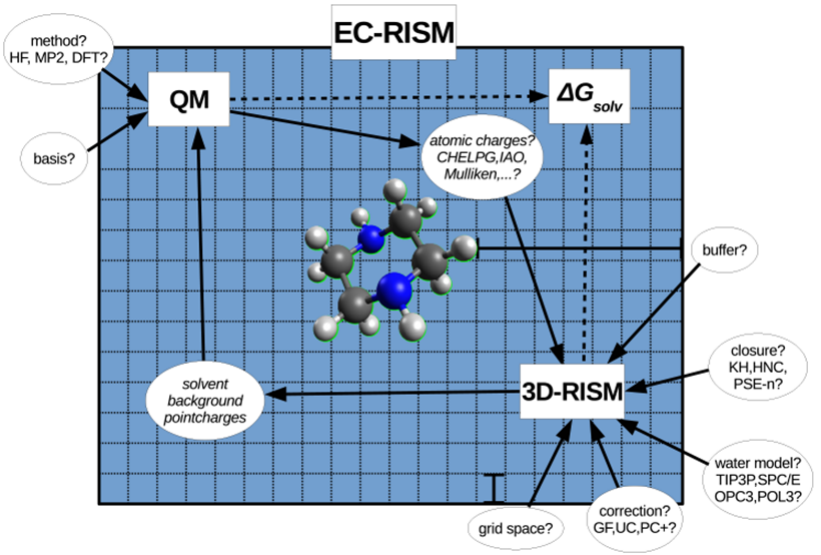

In this work, we implemented the embedded cluster reference interaction site model (EC-RISM) originally developed by Kloss, Heil, and Kast (*J. Phys. Chem. B***2008**, 112, 4337–4343). This method combines quantum mechanical calculations with the 3D reference interaction site model (3D-RISM). Numerous options, such as buffer, grid space, basis set, charge model, water model, closure relation, and so forth, were investigated to find the best settings. Additionally, the small point charges, which are derived from the solvent distribution from the 3D-RISM solution to represent the solvent in the QM calculation, were neglected to reduce the overhead without the loss of accuracy. On the MNSOL[a], MNSOL, and FreeSolv databases, our implemented and optimized method provides solvation free energies in water with 5.70, 6.32, and 6.44 kJ/mol root-mean-square deviations, respectively, but with different settings, 5.22, 6.08, and 6.63 kJ/mol can also be achieved. Only solvent models containing fitting parameters, like COSMO-RS and EC-RISM with universal correction and directly used electrostatic potential, perform better than our EC-RISM implementation with atomic charges.

## Introduction

Most processes take place in solution therefore the solvent effect has to be considered during theoretical approaches if appropriate description of the system is desired. Not only reactions, but also several physicochemical properties are connected to solvation, such as p*K*_a_, dissociation and complexation constants, solvation free energies, log *D*/log *P*, solubilities, and so forth.^1^

One way to determine these properties is to use quantitative structure–property relationships (QSPR), which are usually empirically parametrized functions that relate a target property to a set of molecular descriptors calculable from a simple computational representation of the molecule.^2^ Even though it is a cheap method, this approach lacks the ability to predict accurate values for species which differ considerably from the training data and does not provide insight into the solvation processes.

The other way to predict the physicochemical properties of solvation is to use a solvation model together with the appropriate theoretical computational approach. Solvent molecules can then be treated explicitly which can give a realistic picture of the solvation processes. Because a large number of solvent molecules are needed, this approach is used with molecular dynamics (MD) or Monte Carlo methods (MC), and it is still computationally expensive.^3,4^

In contrast, implicit solvent models treat the solvent as a homogeneously polarizable medium. This greatly reduces the computational costs and enables the usage of quantum mechanical methods in the solvent phase. There are numerous implicit solvent models [polarizable continuum model (PCM),^5^ integral equation formalism PCM (IEFPCM),^6,7^ conductor-like PCM (CPCM),^8^ conductor-like screening model (COSMO),^9^ COSMO for realistic solvation (COSMO-RS),^10^ direct COSMO-RS (DCOSMO-RS),^11^ solvation model based on charge density (SMD),^12^ and so forth], which are discussed elsewhere in detail.^13^ However, these methods lack the ability to describe specific interactions between the solute and solvent.

Another class of solvation approaches is based on integral equation theory (IET). It can provide information about the solvent density around the solute without the need of simulating thousands of solvent molecules, leading to a cost comparable to implicit solvent models. Due to these features, remarkable development has been made in recent years regarding methods exploiting IET, like the reference interaction site model (RISM)^14−16^ or molecular density functional theory (MDFT).^17−22^ About its theory and the various methods, the interested reader is referred to the review of Ratkova et al.^1^ Currently, the most successful formulation of IET is the three-dimensional RISM (3D-RISM) approach.^15,16^ However, there are still challenges concerning the implementation of this model. First, one needs to find the so-called bridge function to solve the RISM equations. There exist no exact functions, but several applicable closure functions have been proposed for that purpose, like the hypernetted-chain (HNC),^23,24^ the Kovalenko–Hirata (KH),^25,26^ and the partial series expansion of order *n* (PSE-*n*) of HNC closure^27^ approaches. Second, the solvation free energies supplied by the theory are far from the experimental ones. This problem is usually treated with a correction after the calculation based on the partial molar volume of the solute, which is already available from the 3D-RISM solution, either by an empirical approach like the universal correction (UC)^28^ or based on theoretical considerations like the pressure correction (PC and PC+) approximation.^29−31^

Because of the attractive features of IET, there were several attempts to combine it with the QM treatment of the solute. Ten-no et al. coupled the Hartree–Fock (HF) self-consistent field (SCF) method with the RISM theory (RISM-SCF),^32^ which was later further improved by Yokogawa et al. with the RISM-SCF-SEDD approach.^33^ Extensions of these methods to 3D-RISM have also been developed.^34−36^ All of these methods solve the RISM equations in every SCF iteration step with partial charges or electron density from the current SCF step providing the electrostatic potential on solute molecules for the next SCF step. Kloss, Heil, and Kast chose another approach for the coupling of QM and RISM called embedded cluster reference interaction site model (EC-RISM).^37^ In this case, the QM solution provides partial charges of the solute to solve the 3D-RISM equations, which gives background point charges describing the solvent for the next cycle of QM calculation. The main advantages are that the QM code and the RISM solver (like AmberTools^38^) can be easily combined, and the self-consistent approach converge well. In the past decade, EC-RISM has been further developed to use exact electrostatics instead of atomic charges, and used successfully in the SAMPL challenges to predict solvation free energies, and p*K*_a_, log *D*, and log *P* values.^39−43^

Here, we report the implementation of the EC-RISM approach with atomic charges of Kloss, Heil, and Kast^37^ and optimize the various RISM and QM settings regarding solvation free energies. We compare our results to other solvation models using the MNSOL^44^ and FreeSolv databases.^4,45^

## Methods

### Theory

#### From IET to 3D-RISM

The RISM theory was discussed in detail elsewhere,^24,46,47^ here, we just provide a brief outline. The starting point of IET is the Ornstein–Zernike (OZ) equation,^48^ which defines the total correlation function *h*(*r*) between a pair of spherical particles:
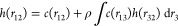
1where ρ is the density of the homogeneous isotropic fluid, *c*(*r*) is the direct correlation function. The pair density distribution function (also known as the pair correlation or pair distribution function) *g*(*r*) can be written as *h*(*r*) + 1. According to the OZ equation, the total correlation between two particles is the sum of the direct correlation function and an indirect correlation function [γ(*r*_12_) = *h*(*r*_12_) – *c*(*r*_12_)], which consists of an infinite series of direct correlations.

The molecular OZ (MOZ) equation is a generalization of the OZ equation to nonspherical molecules.^24,49^ Here, the correlation functions are also dependent on the orientations of the molecules, leading to 6 dimensional equations, making the usage of MOZ nonpractical.

On the basis of the work of Chandler and Anderson,^14^ RISM methods have emerged that reduce the high dimensionality of the MOZ equations. In the one-dimensional RISM (1D-RISM) approach, one-dimensional integral equations and intermolecular spherically symmetric site–site correlation functions are used. The spherical symmetry results in correlation functions that only depend on the *r* distance between sites as
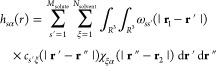
2where *s* refers to the solute sites, whereas α and ξ to the solvent sites. The structures of the molecules are described by the intramolecular correlation function, ω(*r*), while χ_*ξα*_(*r*) is the reduced bulk solvent susceptibility function, and *M*_solute_ and *N*_solvent_ are the number solute and solvent sites. There are several different realizations of 1D-RISM, such as the extended RISM (XRISM),^50^ ARISM^51^ (“A” refers to a scaling coefficient), and dielectrically consistent RISM (DRISM).^52^ The work of Beglov and Roux, and Kovalenko and Hirata introduced the three-dimensional extension of RISM, 3D-RISM.^15,16,53^ In this case, the MOZ equation is replaced by a set of 3D integral equations through partial integration over the orientational coordinates. This leads to the following equations with intermolecular solvent site-solute total correlation functions, *h*_α_(r), and direct correlation functions, *c*_α_(r), which are more feasible:
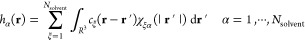
3The reduced solvent susceptibility function can be calculated from a 1D-RISM calculation by
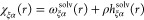
4The solvent susceptibility function can be obtained by multiplying χ with ρ.

As mentioned above, the solution of OZ and RISM requires a so-called closure relation because we have more unknown functions than equations:^24,46,49^
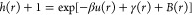
5where *B*(*r*) stands for the bridge function, which is a functional of the indirect correlation function, β = 1/*k*_B_*T*, *k*_B_ denotes the Boltzmann constant, and *T* is the absolute temperature. *u*(*r*) stands for the pair interaction potential, which is usually described by the Coulomb and Lennard-Jones interactions in the RISM formalism. Unfortunately, the exact expression of the bridge function is unknown, so approximations are needed.

The simplest closure is HNC, where the *B*(*r*) term is simply ignored [*B*(*r*) = 0],^54^ however, this closure often leads to convergence issues. Kovalenko and Hirata introduced a partially linearized version of the HNC closure (PLHNC):^26^
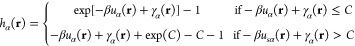
6If *C* = 0, the PLHNC closure becomes the Kovalenko–Hirata (KH) closure. Kast and Kloss used partial series expansion of order *n* (PSE-*n*) of the HNC closure to tackle the convergence problems of HNC:^27^
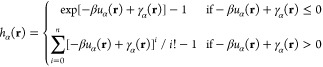
7

Relying on the correlation functions from the solution of 3D-RISM equations, we can determine the solvation free energy (Δ*G*_solv_) using Kirkwood’s equation.^55^ The corresponding free energy functionals for the above-mentioned closures are
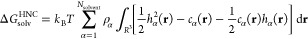
8
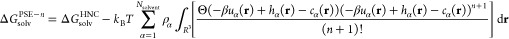
9
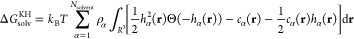
10where ρ_α_ is the number density of solvent site α, and Θ denotes the Heaviside step function:
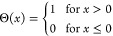
11

Unfortunately, the accuracy of solvation free energies obtained by the 3D-RISM method is not satisfactory. One of the first models to solve this problem assumes Gaussian fluctuations of the solvent molecules (GF) and evaluates the solvation free energy as^56,57^
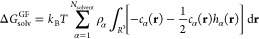
12One of the most successful correction approaches utilizing this approximation is the universal correction (UC).^28^ This model is based on the observation that the solvation free energy errors are linearly dependent on the dimensionless partial molar volume (DPMV, *ρ V̅*) of the solvents,^58^ which can also be determined from 3D-RISM. Therefore, a linear fit on experimental data can produce the fitting parameters (*a*_1_^GF^, *a*_0_^GF^), and the solvation free energy is augmented by a correction as
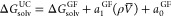
13This expression can be used with any free energy functional not only with GF. Theoretically more established models are the pressure correction (PC) and advanced pressure correction (PC+),^29−31^ which employ the following expressions:
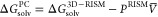
14
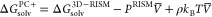
15where *P*^RISM^ is the pressure calculated from the solvent–solvent direct correlation function as
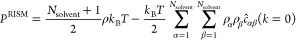
16where *ĉ*_*αβ*_(*k* = 0) is the Fourier transformed direct correlation function at *k* = 0.

#### EC-RISM

RISM as a solvation model can be combined with QM and MM methods. In this work, we focus on the EC-RISM method of Kast and co-workers,^37,59^ which was adopted using the Mrcc quantum chemistry program^60^ for the QM calculations and the AmberTools MD package^38^ for the 3D-RISM calculations. Here, we briefly present the theory behind EC-RISM, which is already explained in detail in the original EC-RISM articles.^37,59^

In this framework, the solvation free energy from 3D-RISM is corrected with the electronic energy change of the solute due to the solvent (Δ*E*^QM^ = *E*_solv_^QM^ – *E*_gas_^QM^):
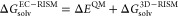
17In this approach, where the solvent is represented as background point charges in the QM calculations, the Hamiltonian of the solute can be written as
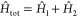
18
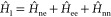
19
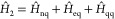
20where *n*, *e*, and *q* refer to the interaction between the nuclei, electrons, and point charges as appropriate.

The energy of the solute, whose wave function (ψ_tot_) is affected by the background charges, can be written as
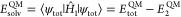
21Therefore, we have to subtract all point charge related energy terms, collected in *E*_2_^QM^ as
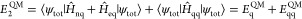
22from the total energy. Here, *E*_qq_^QM^ is the self-energy of the background point charges, and its calculation can be omitted due to the fact that it is also included in *E*_tot_^QM^, and its computation can be demanding when dealing with hundreds of thousands or millions of point charges. *E*_q_^QM^ is the electrostatic interaction energy of the point charges with the solute, which is already taken into account during the 3D-RISM calculations. It can be described as the interaction of the electrostatic potential of the solute (φ) and the charge density ρ_*q*_ at spatial points **r**, which can be simplified to the summation of the products of the background charges [*q*(**r**_*i*_)] and the electrostatic potential at each grid point:
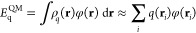
23The required background point charges can be derived from the distribution functions *g* from the 3D-RISM solution by taking the sum of the products of its value [*g*_γ_(**r**_*i*_)] and the charge of the corresponding site (*q*_γ_):
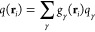
24The 3D-RISM calculation requires the electrostatic potential for the Coulombic interaction of the interaction potential (*u*), which is represented with atomic charges based on the QM calculation. The rest of the input for 3D-RISM is independent of the QM part.

**Figure 1:**
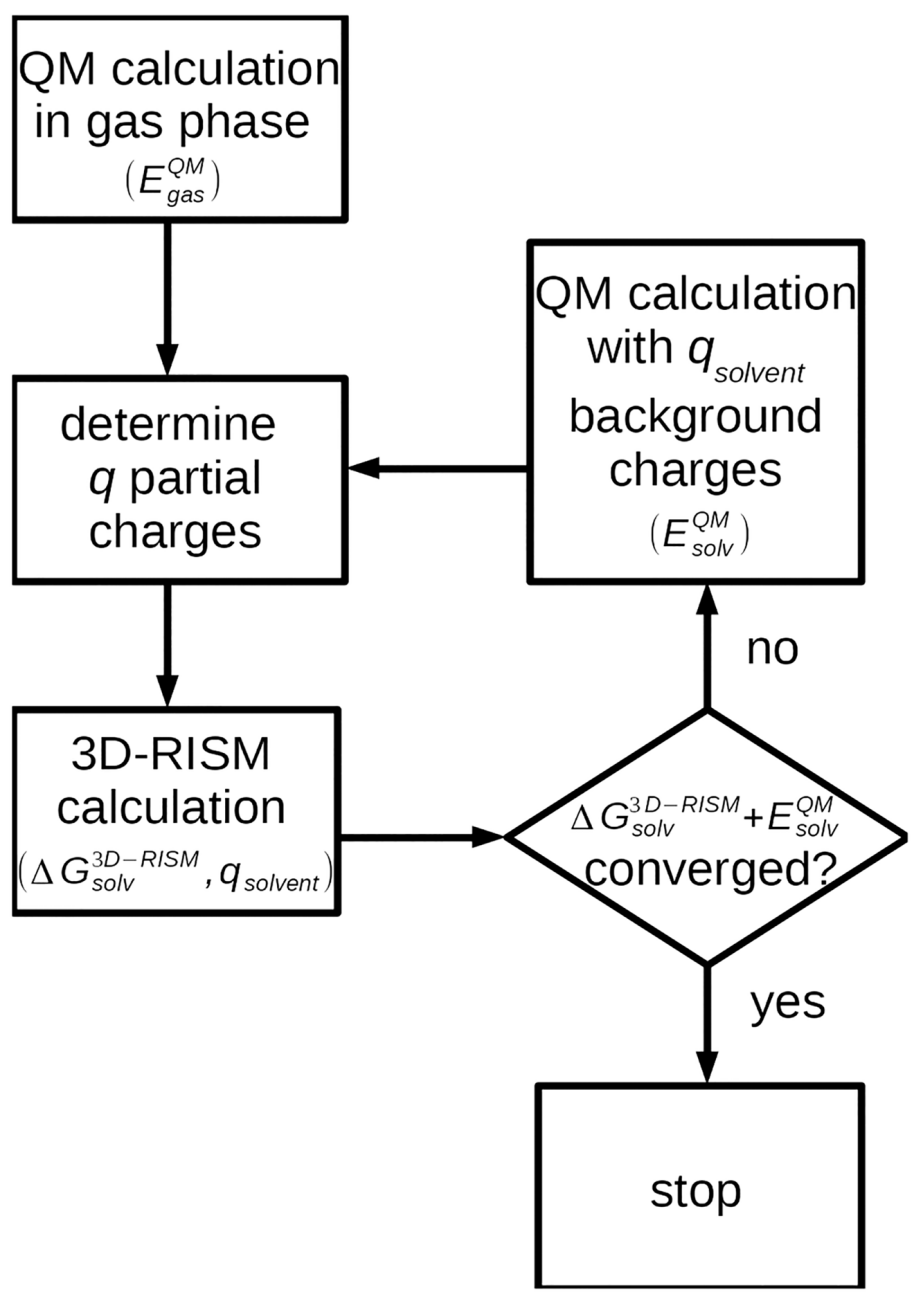
Flowchart of our EC-RISM workflow.

The general workflow of the EC-RISM algorithm is presented in Figure 1. The procedure is initialized by a QM calculation in gas phase. Then, the following steps are repeated until self-consistency, that is, the energy change in QM and RISM is below 0.01 kJ/mol: (i) the solute atomic charges are determined based on a QM calculation, (ii) a 3D-RISM calculation is carried out using the QM atomic charges, and (iii) a QM calculation is performed utilizing the solvent charge distribution obtained from the 3D-RISM solution as background point charges representing the solvent. We note that, instead of using atomic charges to represent the electrostatic potential in the 3D-RISM calculations, the electrostatic potential could also be used directly.^39−41^ The algorithm has been implemented in Mrcc using the AmberTools package for the 3D-RISM calculations. Initial work was aided by the python script of Misin et al.^30,61^

EC-RISM has been used in the SAMPL challenges to predict solvation free energies, and p*K*_a_, log *D*, and log *P* values.^41−43,59,62^ Besides that, the EC-RISM approach has been utilized successfully to study the effect of pressure on various properties,^63^ such as the pressure response of the dipole moment of trimethylamine-*N*-oxide (TMAO) in aqueous solution,^64^ the pressure dependence of NMR chemical shifts for *N*-methylacetamide,^65^ to understand the pressure-induced blue-shift of IR bands of TMAO,^66^ and to develop a force field for urea in aqueous solutions under high pressure.^67^

### Computational Details

As a reference for experimental solvation free energies, the MNSOL^44^ and the FreeSolv^4^ databases were used in this work. We employed the MNSOL[a] data set, which is a subset of the whole MNSOL database and the most commonly used data set for testing and optimizing solvation models. Conformers were generated with openbabel(68) and optimized at the B3LYP/def2-TZVP level of theory with Mrcc in gas phase without relaxing in solution phase. During the optimization of the EC-RISM settings, only the most stable conformers were used with the MNSOL[a] data set, while for the final analysis conformers with energies of at most 10 kJ/mol higher than the most stable one were considered with the MNSOL and FreeSolv databases. The conformationally averaged results were obtained using Boltzmann averaging.

For the QM part of the EC-RISM calculations, various settings were considered. As QM methods, HF, second-order Møller-Plesset (MP2), and several DFT functionals were utilized applying the density-fitting (DF) approximation. Concerning the atomic orbital basis sets, the correlation consistent (aug-)cc-pV*X*Z^69−71^ and def2^72^ bases were tested. Atomic charges are derived from the density of the method used, for example, MP2 density is applied to generate atomic charges if MP2 calculations are performed.

Water susceptibility functions were prepared with the 1drism program of AmberTools^38^ invoking the DRISM theory.^52^ The density was set to 55.343 mol/dm^3^, the dielectric constant to 78.375, and the grid spacing to 0.025 Å. The water models utilized and their parameters are collected in Table 1. The original SPC/E,^73^ TIP3P,^74^ OPC3^75^ and POL3^76^ water models are not sufficient, because the Lennard-Jones parameters of H are needed to avoid the convergence issues of 1D-RISM. Pettitt and Rossky used σ_H_ = 0.4 Å and ϵ_H_ = 0.192464 kJ/mol,^77^ while Hirata and co-workers applied σ_H_ = 1.0 Å and ϵ_H_ = 0.2282372 kJ/mol.^78^ Kast et al. employed σ_H_ = 1.0 Å and ϵ_H_ = 0.234304 kJ/mol for their modified SPC/E water model^59^ (mSPC/E) based on the work of Maw et al.^79^ In this work, we opted for the approach of Luchko et al.,^80^ who proposed the  and ϵ_H_ = 0. 1ϵ_O_ rules. Their modified SPC/E and TIP3P model is referred to as coincident SPC/E (cSPC/E) and coincident TIP3P (cTIP3P). The modified OPC3 and POL3 models are produced following the same approach and will be called cOPC3 and cPOL3. Table S1 of the Supporting Information (SI) presents the compressibilities obtained, and the PC and PC+ correction factors employed in this work.

**Table 1:** Parameters of the Water Models Employed in This Work

	cSPC/E	cTIP3P	cOPC3	cPOL3	mSPC/E
*r*_OH_ (Å)	1.0000	0.9572	1.0000	0.9789	1.0000
θ_HOH_ (deg)	109.47	104.52	109.47	109.47	109.47
*q*_O_ (*e*)	–0.8476	–0.8340	–0.7300	–0.8952	–0.8476
*q*_H_ (*e*)	0.4238	0.4170	0.3650	0.4476	0.4238
σ_O_ (Å)	3.1658	3.1507	3.2037	3.1743	3.1658
σ_H_ (Å)	1.1658^a^	1.2363^a^	1.2037^a^	1.2165^a^	1.0000
ϵ_O_ (kJ/mol)	0.64978	0.63597	0.65270	0.68369	0.64978
ϵ_H_ (kJ/mol)	0.064978^b^	0.063597^b^	0.065270^b^	0.068369^b^	0.234304

a Calculated using .^80^

b Calculated using ϵ_H_ = 0. 1ϵ_O_.^80^

For the 3D-RISM calculations, the rism3d.snglpnt program of AmberTools was utilized with various closure, buffer, grid space, and water models. The GAFF2 force field was used in every calculation. During the work, the temperature was set to 298.15 K where it was necessary. The partial molar volume, which is needed for some corrections, is calculated with the following formula in rism3d.snglpnt:
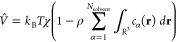
25The parameters for Si were adopted from the work of Dong et al.: a van der Waals radius of 1.778 Å and a well-depth of 0.015 kcal/mol were applied.^81^

## Results and Discussion

### Optimization of the Settings for EC-RISM

As the first step, we optimized those settings that can be arbitrarily improved. These are the buffer and grid space for the RISM calculations and the basis set for the QM calculations. As test molecules, we chose a medium-sized druglike species, piperazine, and a smaller more polar one, NH_3_. The results for piperazine are discussed here, whereas those for NH_3_ are presented in Table S2. In general, there is no significant difference between the conclusions which can be drawn for the two test systems. Other settings, like the used charge model, water model, closure, and correction, cannot be optimized without references because, in contrast to the aforementioned settings, there is no theoretical limit that could provide a reference. Therefore, these are optimized by taking the MNSOL[a] data set, and we compare the results to experimental references.

#### Buffer and Grid Space

The buffer is the distance between the solute and the side of the simulation box, therefore it determines the dimensions of the simulation box. The grid space is practically the distance between the grid points. Larger buffer and smaller grid space obviously improve the accuracy of RISM calculations but increase the cost. To optimize them, piperazine was used as a test molecule using the PSE-3 closure with the PC+ correction and the cSPC/E water model using buffers of 5, 10, 15, ..., 50 Å and grid spaces of 0.1, 0.2, 0.3, 0.4, and 0.5 Å. The results can be seen in Table 2. It can be observed that, with a buffer of 5 Å, the RISM solvation free energy differs at least with 0.1 kJ/mol from that obtained with larger buffers. Out of the tested grid spaces, only the 0.5 Å results differ from the smaller buffers with at least 0.1 kJ/mol (0.3–0.4 kJ/mol). However, using a more polar molecule, such as NH_3_, the results with grid spaces of 0.4 Å differ from the smaller ones with 0.08–0.11 kJ/mol. Therefore, we chose a buffer of 15 Å and a grid space of 0.3 Å for the RISM calculations as a compromise between accuracy and cost.

**Table 2:** Comparison of 3D-RISM Solvation Free Energies (in kJ/mol) and Runtimes (in s) with Different Buffer and Grid Space for Piperazine with PSE-3 Closure, cSPC/E Water Model, and PC+ Correction

grid space (Å)	0.1		0.2		0.3		0.4		0.5	
buffer (Å)	Δ*G*_solv_	time	Δ*G*_solv_	time	Δ*G*_solv_	time	Δ*G*_solv_	time	Δ*G*_solv_	time
5	–36.01	41.2	–35.96	4.2	–35.88	1.3	–35.81	0.5	–35.40	0.3
10	–36.11	175.1	–36.07	21.5	–36.09	6.3	–36.07	2.1	–35.71	1.2
15	–36.11	494.5	–36.07	64.7	–36.10	19.8	–36.07	6.2	–35.72	3.2
20	–36.12	959.8	–36.07	123.2	–36.11	38.9	–36.08	13.2	–35.73	7.0
25	–36.12	1960.6	–36.07	240.2	–36.11	78.9	–36.08	26.5	–35.73	14.1
30	–36.13	3112.4	–36.07	389.1	–36.11	120.0	–36.09	44.7	–35.73	26.8
35	–36.13	4758.8	–36.07	608.3	–36.11	173.9	–36.09	70.7	–35.73	35.4
40			–36.06	863.0	–36.11	276.9	–36.09	104.1	–35.73	62.5
45			–36.03	1235.9	–36.11	356.5	–36.09	135.5	–35.72	73.1
50			–36.02	1774.7	–36.08	468.8	–36.06	207.6	–35.71	94.7

#### Cost Reduction by Dropping Small Charges

**Figure 2:**
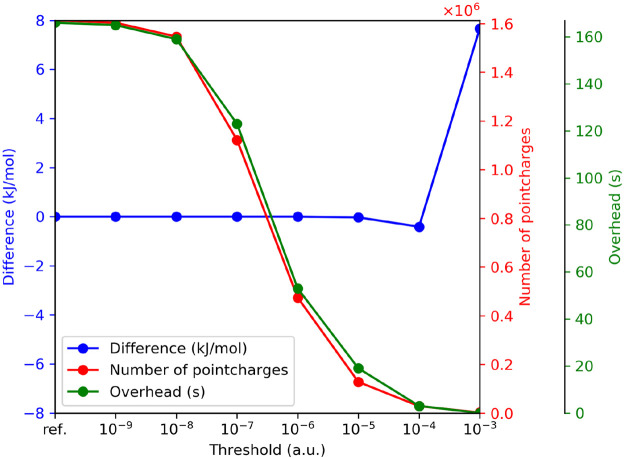
Effect of neglecting background point charges below a threshold on the energy and the runtime. The reference is a calculation where there are no point charges dropped from the RISM calculation with applying a 15 Å buffer, 0.3 Å grid space, cSPC/E water, PC+ correction, and PSE-3 closure. For comparison, the DF-MP2/def2-TZVPPD gas-phase calculation of piperazine requires 101 s on an Intel(R) Xeon(R) CPU E5–1650 v2 @ 3.50 GHz processor with 12 cores.

The treatment of the millions of point charges generated during the 3D-RISM calculation can be cumbersome. However, most of them are too small and/or too far from the molecule to have an impactful effect on the QM results. Therefore, we can drop these insignificant point charges to speed up the QM calculations. Figure 2 shows the results for the first QM calculations of the EC-RISM cycle by dropping point charges below a certain threshold from the first RISM calculation with a buffer of 15 Å, grid space of 0.3 Å, cSPC/E water, PC+ correction, and PSE-3 closure. It can be seen that charges smaller than 10^–6^ a.u. can be neglected without any loss of accuracy, which results in dropping 70.6% of all point charges in this setting. Also, the overhead due to the background point charges is proportional to the number of point charges. Even more point charges can be dismissed with thresholds of 10^–5^ or 10^–4^ a.u., leading to a noticeable but acceptable difference of a few hundredths or tenths of a kJ/mol. If a larger or denser grid is used for the RISM calculation, then an even bigger speedup can be achieved with this technique. With the more polar NH_3_, similar results are obtained. From now on, we use the conservative threshold of 10^–6^ a.u. for dropping insignificant charges.

#### Basis Set Dependence

**Figure 3:**
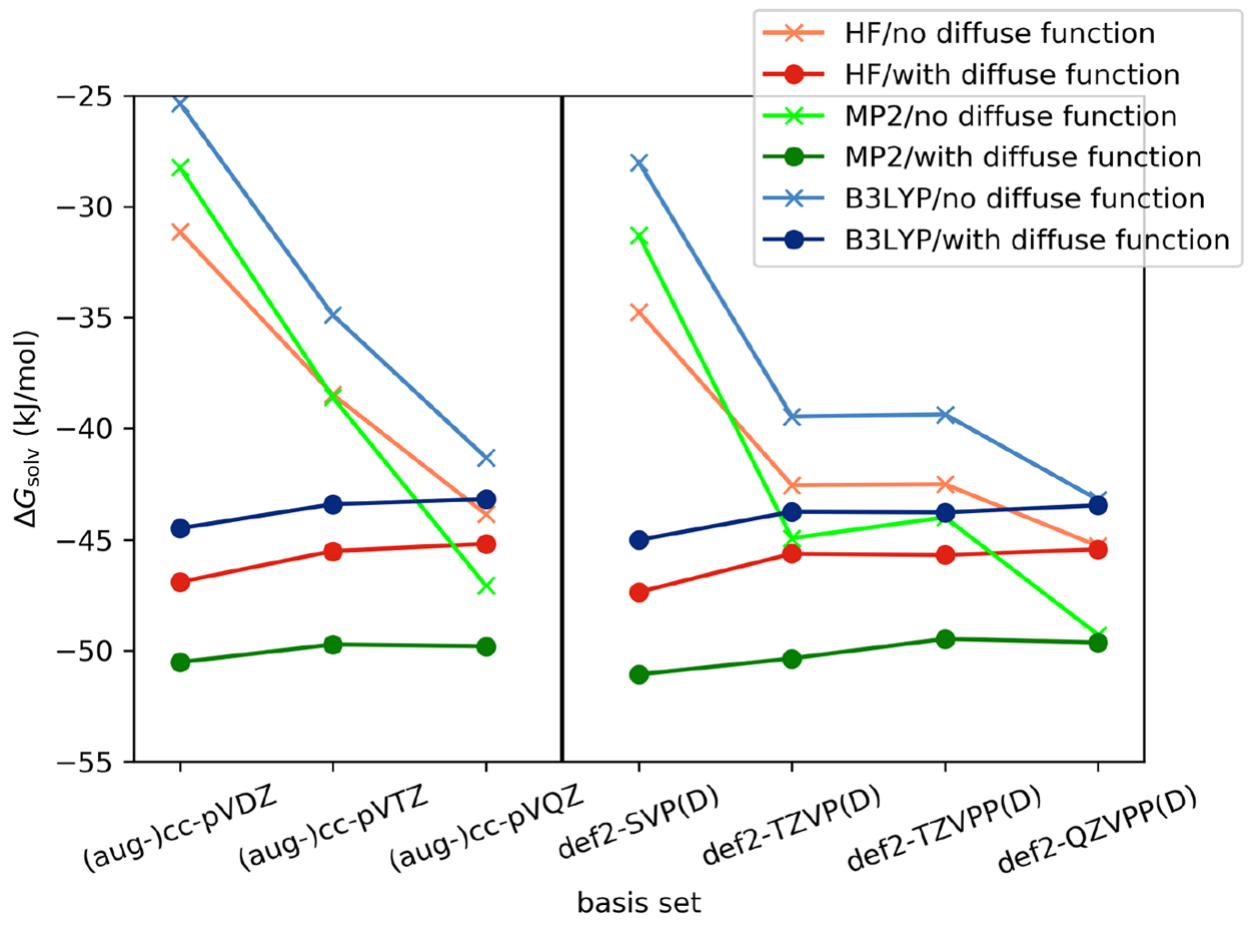
Effect of the basis set on the EC-RISM solvation free energies of piperazine using PSE-3 closure, PC+ correction, cSPC/E water, 15 Å buffer, and 0.3 Å grid space with CHELPG charges.

The size of the basis sets can also be arbitrarily increased until we reach the basis set limit, so it is clear that this setting should also be investigated. In Figure 3, we compared the EC-RISM solvation free energies of piperazine using the previously determined settings along with the PSE-3 closure, cSPC/E water, and PC+ correction, with various basis sets of double- (DZ), triple- (TZ), and quadruple-ζ (QZ) quality at the HF, MP2 and B3LYP levels of theory. For the DZ and TZ basis sets, we can see a clear difference between the results of bases with and without diffuse functions. The most notable difference, 22.3 kJ/mol, can be observed between the MP2/cc-pVDZ and MP2/aug-cc-pVDZ results. If diffuse functions are used, the solvation free energies become almost independent of the size of the basis set. However, without diffuse functions, the EC-RISM results are heavily dependent on the basis set size, and only QZ-quality bases can approach the performance of basis sets with diffuse functions. Therefore, diffuse functions are necessary for accurate results. Even DZ-quality basis sets with diffuse functions can provide acceptable results, but the TZ-quality bases sets are preferred. Again, for the more polar NH_3_, the conclusions are similar, see Table S2. For further calculations in this work, the def2-TZVPPD basis set is employed as this basis provides almost converged solvation energies and is somewhat more economical than the aug-cc-pVTZ basis, which is of similar quality.

#### Charge Models and Corrections

Here, the effect of various charge models (CHELPG,^82^ Merz-Kollman,^83^ IAO,^84^ Mulliken,^85^ Löwdin^86^) and 3D-RISM corrections (PC, PC+, Gaussian fluctuation, UC) on the quality of the computed solvation free energies is tested. The charge model is needed to determine the Coulomb interaction of the solute and the solvent for the rism3d.snglpnt program. As mentioned earlier, the corrections are essential to get solvation free energies which are comparable to the experimental ones.

Table 3 shows the RMSDs of the EC-RISM solvation free energies against the MNSOL[a] reference values with the different charge models and corrections. The QM part was calculated at DF-MP2/def2-TZVPPD level of theory, while the PSE-3 closure with the cTIP3P water model was applied in the RISM calculations. With the Löwdin and Mulliken charges, respectively, 54 and 32 out of 273 species not converged, therefore they are not recommended. Also the accuracy of the converged ones are way worse than with the other charges with the RMSDs being above 20 kJ/mol. In line with the previous experience,^87^ the electrostatically fitted charges, that is, CHELPG and MK, perform better than the other charge models studied. Out of the four corrections, the theoretically derived PC+ and the fitted UC perform similarly, but obviously UC is slightly better. Consequently, as a charge model, CHELPG and MK are recommended, while PC+ and the universal correction are preferred to determine solvation free energies. These setting together provide RMSDs between 5 and 7 kJ/mol. We prefer PC+ because it does not require the determination of fitting parameters for different settings, even though it can be somewhat less accurate.

**Table 3:** Effect of Various Charges and Corrections on the RMSDs of Solvation Free Energies on the MNSOL[a] Dataset (Values in kJ/mol)^a^

corrections	CHELPG	MK	IAO	Mulliken^b^	Löwdin^c^
PC	15.08	17.05	19.28	*34.00*	*45.19*
PC+	5.75	6.40	10.76	*26.56*	*41.68*
UC	5.41	5.16	9.92	*20.21*	*41.30*
GF	21.66	21.27	19.73	*29.88*	*88.55*

a Values in italic are only informative but not directly comparable to non-italic ones.

b 54 out of the 273 species are not converged.

c 32 out of the 273 species are not converged.

The use of atomic charge models can be bypassed by employing the solute electrostatic potential computed by the QM approach directly in the 3D-RISM calculation as it was suggested by Frach and Kast,^40^ but we have not yet implemented this approximation.

Hereafter, CHELPG charges and the PC+ correction will be utilized.

#### Water Models and Closures

**Figure 4:**
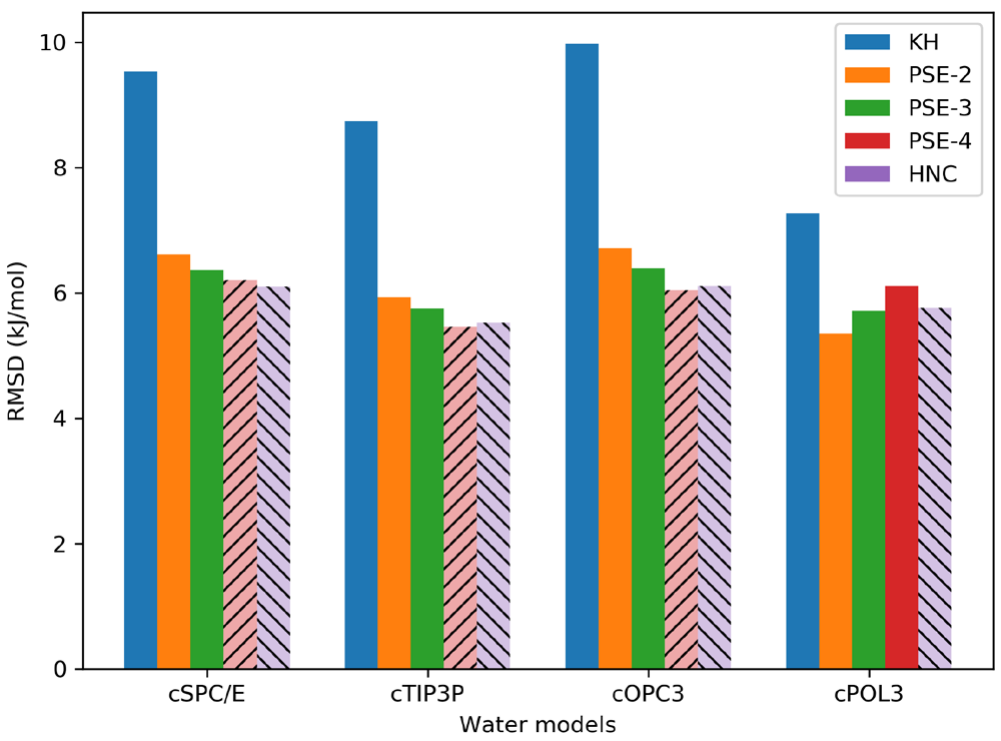
Comparison of RMSDs on the MNSOL[a] subset for different water models and closure relations. The paled and hatched bars represent the convergence issues with that pairing.

The closure function and the water/solvent model are other settings in RISM calculations where we have several options. Figure 4 displays our results on the MNSOL[a] data set with different closure functions (KH, PSE-2, PSE-3, PSE-4, HNC) and various water models (cSPC/E, cTIP3P, cOPC3, cPOL3). We experienced convergence issues with the PSE-4 and HNC closures, which were also discussed in the literature.^1^ The use of PSE-2 and PSE-3 is a good compromise between accuracy and convergence performance, while the KH closure provides results with a larger RMSD than the other closures, around 7–10 kJ/mol. The water models perform similarly yielding RMSDs of around 5–7 kJ/mol with the PSE-2 and PSE-3 closures. The best combinations are PSE-3/cTIP3P and PSE-2/cPOL3. Another observation is that the standard deviation of the differences is rather similar across the board, 5.3–5.8 kJ/mol, and these settings just shift the solvation free energies but do not change them fundamentally. From KH to HNC, the solvation free energy increases, while the increasing order for water models is cOPC3 < cSPC/E < cTIP3P < cPOL3. More detailed statistics and results can be found in Table S3.

In summary, any of the studied water models can be used along with the PSE-2 or PSE-3 closure to get accurate solvation free energies. The PSE-3/cTIP3P and PSE-2/cPOL3 pairings are used in the next section to compare the method to other solvation models.

#### Level of Theory of QM Calculation

Besides MP2, HF, and several DFT functionals of different rungs (revPBE,^88^ SCAN,^89^ ω-B97X-V,^90^ B2GPPLYP^91^) were also tested. Table 4 shows the corresponding statistics on the MNSOL[a] data set. HF performs worse than MP2 and DFT with and RMSD of 9.34 kJ/mol. The performance of the various DFT functionals is fairly similar with the RMSD ranging from 6.3 to 6.8 kJ/mol, which is worse than MP2 but still acceptable. Somewhat surprisingly, the more sophisticated double hybrid functional is also outperformed by MP2. This shows that the EC-RISM method is robust regarding the level of theory.

**Table 4:** Error Statistics Using HF, MP2, and Various DFT Approaches as the QM Method in EC-RISM with PSE-3 Closure, cTIP3P Water Model, PC+ Correction, and def2-TZVPPD Basis Set on the MNSOL[a] Dataset

statistics	HF	MP2	revPBE	SCAN	ω-B97X-V	B2GPPLYP
MSD	–5.44	–1.56	0.13	–1.91	–2.60	–2.08
MAD	6.81	4.40	5.58	4.96	4.98	4.84
RMSD	9.34	5.75	6.82	6.33	6.60	6.31

#### Comparison with the Original Implementation

We reproduced the results of the SAMPL6 paper on the MNSOL[a] subset^41^ within reasonably accuracy, knowing the differences between the RISM and CHELPG implementations. The main differences between our and the approach used in the SAMPL6 challenge (with atomic charges) are the following. Here, the QM method is MP2/def2-TZVPPD instead of MP2/6-311+G(d,p). We optimized the geometry in gas phase at the B3LYP/def2-TZVP level of theory, while Kast et al. used B3LYP/6-311+G(d,p) in gas phase and B3LYP/6-311+G(d,p)/IEFPCM in solution phase. They employed CHELPG charges fitted on the HF density and only calculated MP2 energy in the last step, while we take the density of the QM method applied throughout the whole calculation. Also, we used 1.75 Å as a radius for Br to determine CHELPG charges, while Kast et al. applied 1.3 Å. Additionally, they employed HF electrostatics to determine *E*_q_^QM^, while we used the corresponding MP2 electrostatics. For the 3D-RISM computations, we applied a water susceptibility function produced with the same closure that is otherwise utilized in the 3D-RISM calculation, while in the original version, the susceptibility function of mSPC/E water model^79^ was employed with HNC closure. Also we apply PC+ correction, whereas Kast and co-workers utilized an UC-like correction, similar to eq 13, but *a*_0_ was set to 0. For the 3D-RISM calculations, we applied the GAFF2 force field, instead of GAFF used by Kast et al.

Beyond the reproduction of the SAMPL6 results, we compared the effects of the different electrostatics (HF or MP2), basis sets (6-311+G** or def2-TZVPPD), and water model (cSPC/E, cTIP3P, or mSPC/E) on the MNSOL[a] data set using the setup of the Kast group, that is, geometries from the SAMPL6 paper, 1D-RISM with HNC, 3D-RISM with PSE-2, GAFF force field and Br radius set to 1.3 Å. The comparison can be seen in Table 5.

**Table 5:** Error Statistics on the MNSOL[a] Dataset in kJ/mol along with c_*V*_ Correction Factors in kJ/mol/Å^3^ Using Different Settings to Compare Our and the Original SAMPL6 Implementation^a^

			6-311+G**				def2-TZVPPD			
ESP	water model	correction	MSD	MAD	RMSD	c_*V*_	MSD	MAD	RMSD	c_*V*_
HF	mSPC/E	UC	–0.813	4.729	6.359	–0.42977	–0.522	4.328	5.754	–0.43269
HF	mSPC/E	PC+	–5.577	6.458	8.152	–0.46081	–4.846	5.802	7.385	–0.46081
HF	cSPC/E	UC	–0.801	4.789	6.430	–0.49790	–0.509	4.402	5.838	–0.50087
HF	cSPC/E	PC+	–2.890	5.267	6.809	–0.51181	–2.155	4.715	6.099	–0.51181
HF	cTIP3P	UC	–0.699	4.531	5.990	–0.47846	–0.410	4.242	5.512	–0.48141
HF	cTIP3P	PC+	–1.943	4.724	6.136	–0.48673	–1.212	4.339	5.578	–0.48673
MP2	mSPC/E	UC	–0.924	5.463	7.255	–0.41517	–0.618	4.871	6.474	–0.42076
MP2	mSPC/E	PC+	–7.974	8.472	10.468	–0.46081	–6.812	7.399	9.266	–0.46081
MP2	cSPC/E	UC	–0.883	5.529	7.366	–0.48210	–0.617	4.948	6.570	–0.48829
MP2	cSPC/E	PC+	–5.371	6.918	8.797	–0.51181	–4.172	5.935	7.594	–0.51181
MP2	cTIP3P	UC	–0.765	5.067	6.736	–0.46397	–0.500	4.639	6.063	–0.46997
MP2	cTIP3P	PC+	–4.209	6.061	7.680	–0.48673	–3.039	5.201	6.644	–0.48673

a ESP refers to the electrostatics used for the determination of CHELPG charges and E_*q*_ energy. Gas and solvent phase geometries are from the SAMPL6 work of Kast et al.,^41^ which have been corrected.^92^ 1D-RISM calculations for solvent susceptibility file were made with HNC closure, while 3D-RISM calculations with PSE-2 closure. The GAFF force field was utilized, and the radius of Br was set to 1.3 Å.

Obviously, the fitted UC correction performs better than the PC+ correction, as stated earlier. The effect of the basis set is also clear, the larger and more reliable def2-TZVPPD produces better error statistics than 6-311+G** in every case. The water model used by Kast et al. with UC correction and HF electrostatics, that is, the original EC-RISM setup with atomic charges for SAMPL6, provides similar results, RMSDs of 6.359 and 5.754 kJ/mol with 6-311+G** and def2-TZVPPD, respectively, while with the cSPC/E water model, RMSDs of 6.430 and 5.838 kJ/mol are obtained, which are slightly worse statistics than with the cTIP3P water model, RMSDs of 5.990 and 5.512 kJ/mol. However, with PC+ correction, we got more negative solvation free energies with the mSPC/E water model than with the others, leading to worse error statistics. A similar conclusion can be drawn comparing the statistics of MP2 electrostatics of the water models to each other.

Surprisingly, HF electrostatics performs better than MP2 electrostatics on the MNSOL[a] data set. MSD shows that MP2 electrostatics lowers the solvation free energies, which are already lower than the reference on average, and that leads to worse performance. Even though, it seems better to use HF electrostatics, we continue to employ our approach, that is, to use the same electrostatics for the CHELPG charges and the E_*q*_ energy as the QM level of theory because we think that it is problematic to use the interaction energy of the HF density and the point charges to correct the MP2 energy instead of the interaction energy of MP2 density and the point charges. However, the usage of HF electrostatics can save around 30–50% computational time in the intermediate cycles by not calculating the MP2 energy and density, only at the last cycle.

In summary, any of the water models can be used with the UC correction, and the results can be improved with larger basis sets, however, cTIP3P performs better with PC+. Also, based on theoretical considerations, we prefer MP2 electrostatics over HF electrostatics, even though the latter gives better error statistics in every studied setup.

### Comparison with Other Solvation Models

The best two settings, PSE-3/cTIP3P and PSE-2/cPOL3, were tested on both the full MNSOL and the FreeSolv databases with the relevant conformers taken into account, while other settings were kept as determined earlier: MP2/def2-TZVPPD, PC+ correction, 15 Å buffer, 0.3 Å grid space, CHELPG charges. We are aware of the fact that the good performance of a model on these data sets does not always lead to successful prediction of solvation parameters in general, as the SAMPL challenges showed.^93−96^ However, without studying other solvents and ionic species, which will be addressed in future works, we cannot test the method on the SAMPL challenges and have to rely on the MNSOL and FreeSolv data sets.

For the MNSOL[a] database, we got slightly better RMSDs considering the conformers (5.70 and 5.22 kJ/mol) than originally (5.75 and 5.35 kJ/mol). The full MNSOL database gave higher RMSDs with 6.32 and 6.08 kJ/mol. However, for the FreeSolv database PSE-3/cTIP3P performed slightly better: an RMSD of 6.44 kJ/mol was obtained with this combination of settings, while PSE-2/cPOL3 yielded 6.63 kJ/mol.

**Figure 5:**
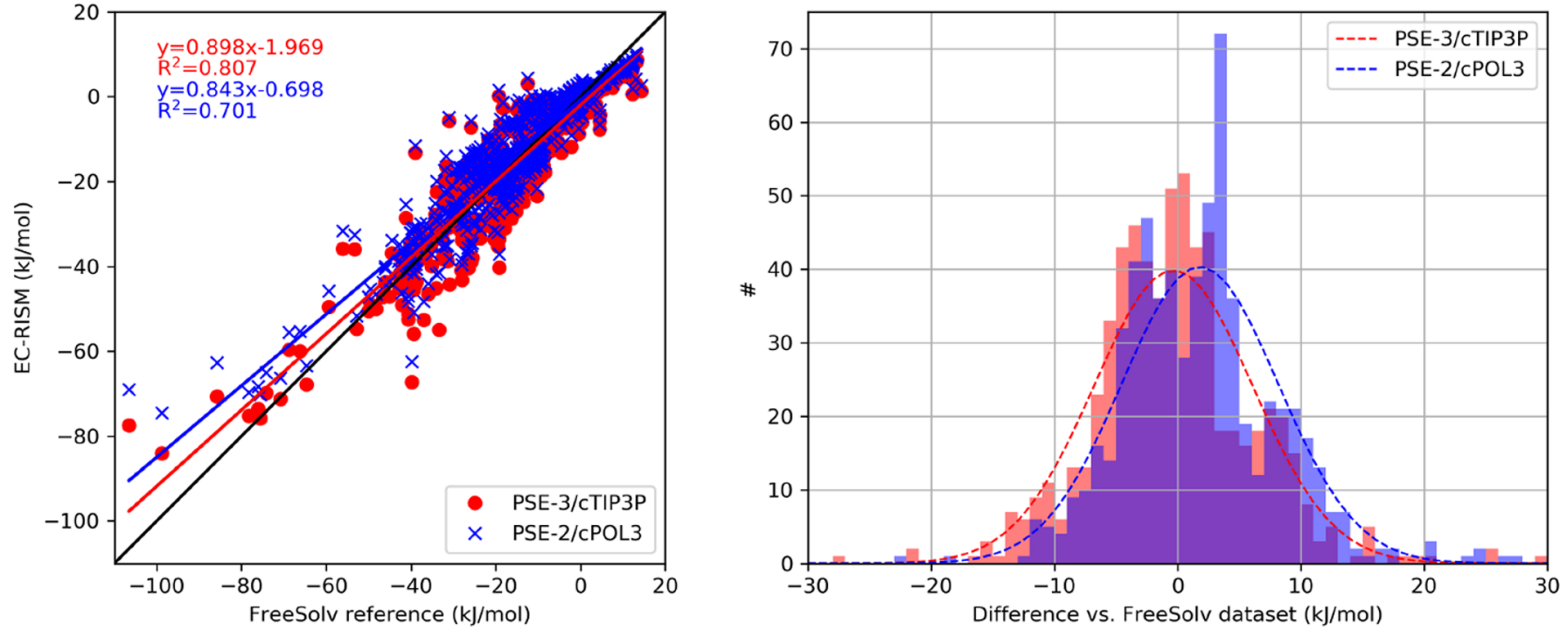
Calculated vs reference experimental solvation free energies (left) and histogram of errors (right) on the FreeSolv data set for PSE-3/cTIP3P and PSE-2/cPOL3 EC-RISM settings.

Figure 5 compares the calculated and reference solvation free energies and presents the histogram of the differences. We can see that larger deviations from the reference values occur for the more negative solvation free energies, while the distribution is close to the desired Gaussian distribution for PSE-3/cTIP3P with the FreeSolv data set. However, for PSE-2/cPOL3 the distribution is skewed, the peak of the histogram is at 3–4 kJ/mol.

The most problematic species can be seen in Table S4. We cannot determine a clear reason why the EC-RISM results deviate from the reference values for the outliers. Some of them, d-xylose, d-glucose, mannitol, and glycerol, have numerous hydroxyl groups, which can be challenging to taken into account properly. Also druglike molecules (nitralin, trichlorfon, flufenamicacid) have several different functional groups. It is surprising that such simple species as 2-nitrophenol and 2-iodophenol can cause difficulties for this method and result in a 20 kJ/mol error. Problems can also arise due to the improper atom type assignment during the force field application. Also, it is possible that the accuracy and reliability of some data is questionable.

We also compared the performance of our EC-RISM method to other solvation models. The RMSDs of the methods on the MNSOL, MNSOL[a] subset, and FreeSolv databases are collected in Table 6, while additional statistics with other methods are listed in Table S5.

**Table 6:** Comparison of RMSDs of Several Solvation Models on the MNSOL[a], MNSOL, and FreeSolv Satasets^a^

method	FreeSolv	MNSOL[a]	MNSOL
EC-RISM, MP2/def2-TZVPPD/			
PSE-3/cTIP3P/CHELPG/PC+, this work	6.44	5.70	6.32
EC-RISM, MP2/def2-TZVPPD/			
PSE-2/cPOL3/CHELPG/PC+, this work	6.63	5.22	6.08
EC-RISM, MP2/6-311+G(d,p)/			
PSE-2/mSPC/E/φ_*opt*_/UC^41^	–	6.19	6.53
EC-RISM, MP2/6-311+G(d,p)/			
PSE-2/mSPC/E/CHELPG/UC^41^	–	6.59	7.40
EC-RISM, MP2/cc-pVTZ/			
PSE-2/mSPC/E/φ_*opt*_/UC^41^	–	4.73	5.44
3D-RISM, PSE-4/cTIP3P/AM1-BCC, this work	7.12	6.33	8.18
3D-RISM, PSE-3/cPOL3/AM1-BCC, this work	7.39	5.92	7.50
MD^4,45^	6.45	–	–
DCOSMO-RS ϵ^11^	–	5.93	–
DCOSMO-RS ∞^11^	–	6.80	–
COSMO^11^	–	10.42	–
COSMO-RS^11,97^	–	3.48	–
SMD, IEF-PCM/G03/M05–2x^12^	–	3.60	–
SMD, M05-2*X*/6-31G(d), this work	5.90	4.20	6.85
IEF-PCM, G03d(UAHF)/HF^12^	–	7.32	–
PCM, M05-2*X*/6-31G(d), this work	9.00	7.95	9.53
C-PCM, G06d/B3LYP^12^	–	7.74	–

a Values are in kJ/mol.

On the MNSOL[a] data set, the COSMO-RS and the SMD solvation models perform the best with RMSDs of under 4 kJ/mol, but it must be noted that they were optimized for this data set. The EC-RISM implementation of Kast et al.,^41^ where, instead of CHELPG charges, the electrostatic potential is used directly in the RISM calculations, is capable of providing an RMSD of 4.73 kJ/mol. This shows that our implementation has still room for improvements.

For a better comparison for SMD, PCM, and 3D-RISM, we performed calculations for the whole FreeSolv and MNSOL databases. For 3D-RISM, we used similar settings as for EC-RISM, but AM1-BCC charges were employed. For SMD and PCM, we used the M05-2*X*/6-31G(d) level of theory. The results are varying for SMD: though it performs slightly worse than the reference on MNSOL[a], it is better than our EC-RISM on the FreeSolv database (RMSDs of 5.90 vs 6.44 kJ/mol) but worse on the whole MNSOL test set (6.85 vs 6.32 kJ/mol).

Comparing our and Kast’s EC-RISM, the former performs better on the whole MNSOL data set, except when the cc-pVTZ basis set and the direct electrostatic potential are utilized. The reason for that is that they use UC which is fitted on the MNSOL[a] data set, while our implementation is free from such empirical parameters. Another difference is that they use HF during the iterations, and MP2 is only employed for the converged state, which can speed up the calculation but can have the opposite effect on the accuracy.

If we look at the pure 3D-RISM, we can see that PSE-3/cPOL3 is the best performing approach on the MNSOL[a] and MNSOL data sets (RMSDs of 5.92 and 7.50 kJ/mol), while PSE-4/cTIP3P is better on the FreeSolv database with an RMSD of 7.12 kJ/mol. That is, 3D-RISM is slightly worse than our EC-RISM but still provides comparable solvation free energies. The COSMO and PCM models are not reliable for the calculation of solvation free energies. The MD results provided by Mobley for their FreeSolv data set show 6.45 kJ/mol as RMSD, which is similar to our 6.44 kJ/mol for our EC-RISM result with PSE-3/cTIP3P.

On the basis of these results, it is hard to say which is the best solvation model, but besides COSMO-RS, our method is definitely comparable with the original EC-RISM of Kast, MD, and SMD solvation models. Even though EC-RISM is more expensive than implicit solvent models due to the fact that it requires 2–8 more QM calculations to converge, RISM can provide additional information about solvent distribution. Further optimization and testing is needed for ionic solutes and nonwater solvents, which will be addressed in future studies.

## Conclusions

In this work, we implemented the EC-RISM method of Kloss, Heil, and Kast^37^ with atomic charges and optimized various settings of it. We have found that the buffer should be at least 10 Å, but we recommend 15 Å. For grid space, we chose 0.3 Å, but it should be less than 0.5 Å. The solvent distribution of RISM is represented as point charges on a grid, and the small point charges can be discarded to reduce the overhead of the QM calculation. For the threshold for neglecting the point charges, we suggest 10^–6^ a.u., but 10^–5^ a.u. is still acceptable. Regarding the basis sets, we found that for EC-RISM calculations, the usage of diffuse functions is recommended, and def2-TZVPPD was utilized throughout the work. As for the charge models, the ones based on the electrostatic potential, like CHELPG and MK, provide acceptable results, and we chose CHELPG as default. Out of the several corrections for 3D-RISM, only PC+ and UC provide meaningful results regarding solvation free energy, and we recommend to use PC+ because it does not require a training set to determine the fitting parameters for other settings. The level of the QM calculation can be almost anything, MP2 is slightly better than other DFT alternatives. The choice of the water model has only a small effect, but cTIP3P and cPOL3 perform better than cSPC/E and cOPC3. The closure relation for 3D-RISM should be PSE-2 or PSE-3 with PSE-3/cTIP3P and PSE-2/cPOL3 being the best pairings. However, PSE-3/cTIP3P has been chosen as default because it performs better on the largest FreeSolv data set, and a cTIP3P water model file is already available in the recent AmberTools versions.

On the MNSOL[a], MNSOL, and FreeSolv databases, we got 5.70, 6.32, and 6.44 kJ/mol RMSDs for the optimized settings with PSE-3/cTIP3P, while PSE-2/cPOL3 produced 5.22, 6.08, and 6.63 kJ/mol, respectively. Compared to other solvation models, COSMO-RS has the best performance on the MNSOL[a] subset (3.48 kJ/mol),^11,97^ but it was optimized on that data set, and we have no data for the other data sets. The EC-RISM of Kast et al. using the electrostatic potential directly and the UC correction also performs better on the MNSOL database than our implementation (RMSD of 5.44 kJ/mol), but with different basis set and/or with CHELPG charges, slightly worse RMSDs, 6.53 and 7.40 kJ/mol, can be obtained. SMD, which is also fitted on the MNSOL[a] subset, provides better statistics on the MNSOL[a] and FreeSolv data sets but worse on MNSOL. Pure 3D-RISM produces acceptable solvation free energies, but the RMSDs are obviously worse then the EC-RISM ones. The performance of MD is comparable to our implemented method. In summary, only solvent models containing fitting parameters, COSMO-RS and EC-RISM with universal correction, perform better than our EC-RISM implementation.

We note that our approach could be improved with the direct usage of the electrostatic potential in the RISM calculations^41^ and also with better force fields, especially with polarizable ones.^39^ The methodology can also be extended to ions, which requires an explicit charge correction term, and nonwater solvents as suggested by the EC-RISM results in the SAMPL challenges,^41^ and this is planned for future work.

## Supporting Information Available

Solvation free energies and statistics on the MNSOL[a], MNSOL, and FreeSolv databases; geometries for all the systems discussed in this paper. Also raw data is provided in csv format including the names of the species in the MNSOL and FreeSolv databeses, reference values, uncorrected 3D-RISM solvation free energies, PMV, corrections to 3D-RISM, corrected 3D-RISM results, gas-phase electronic energies, electronic energies in solution, and the EC-RISM solvation free energies (ZIP)
